# Thermoregulatory and Behavioral Responses of Buffaloes With and Without Direct Sun Exposure During Abnormal Environmental Condition in Marajó Island, Pará, Brazil

**DOI:** 10.3389/fvets.2020.522551

**Published:** 2020-11-25

**Authors:** Letícia Godinho Athaíde, Waleria Cristina Lopes Joset, Jean Caio Figueiredo de Almeida, Messy Hennear de Andrade Pantoja, Rafaella de Paula Pacheco Noronha, Andréia Santana Bezerra, Antônio Vinicius Corrêa Barbosa, Lucieta Guerreiro Martorano, Jamile Andréa Rodrigues da Silva, José de Brito Lourenço Júnior

**Affiliations:** ^1^Institute of Veterinary Medicine, Federal University of Pará, Belem, Brazil; ^2^Institute of Animal Health and Production, Federal Rural University of the Amazon, Belem, Brazil; ^3^Cyberspace Institute, Federal Rural University of the Amazon, Belem, Brazil; ^4^Embrapa Eastern Amazon, Belem, Brazil

**Keywords:** amazon, ambience, ethology, physiology, thermography

## Abstract

This study aimed to assess the effect of thermal-hydraulic variables in female buffaloes with or without direct solar exposure in a year of strong El Niño through behavior responses and infrared thermography to reinforce the environmental comfort indicators, in Marajó Island, Pará, Brazil. The experiment was carried out in Cachoeira do Arari municipality and 20 female Murrah buffaloes were randomly assigned to two groups: Group WS (*n* = 10) was kept in pickets with native trees. Group NS (*n* = 10) was kept in crush squeeze with no shade. Data on air temperature (AT, °C), relative air humidity (RH, %), wind velocity (WV, m/s), rectal temperature (RT), respiratory rate (RR), and body surface temperature (BST) were collected. Practical Buffalo Comfort Climatic Condition Index (BCCCI), practical Buffalo Environmental Comfort Index (BECI), Temperature and Humidity Index (THI) and Benezra's Thermal Comfort Index (BTCI) were obtained. Infrared thermography analysis was carried out with a FLIR T-series T640bx camera. Data on time spent grazing, ruminating, idleness, and in other activities were recorded. A significant difference in AT of ~1°C was found between the groups at 6 a.m., 10 a.m. and 6 p.m. THI indicated emergency conditions. Female buffaloes were at danger PBCCCI conditions at 2 p.m. There was also significant difference for RT between treatments at 10 a.m., 2 p.m. and 6 p.m., whose values were higher (*P* < 0.05) for animals from NS Group, with the highest mean time at 2 p.m. Pearson correlation was significant and positive (*P* < 0.01) between RT mean and VUL, TI and ORB mean, maximum and minimum temperatures. The total time given to grazing was 518.2 min for the group NS and 629.5 min for the group WS. Rumination was more pronounced in the afternoon shift for the group NS. Buffaloes kept in a system with trees graze, ruminate and perform other activities with more intensity than animals raised in systems without access to shade, and tend to hyperthermia, mainly at 10 a.m. and 2 p.m., in Marajó Island, Pará, Brazil.

## Introduction

The Marajó Archipelago, Pará, Brazil, is the largest marine fluvial complex in the world, with an area of 49,606 Km^2^ and 438,694 thousand inhabitants, and has the second largest buffalo population in Brazil, 377,150 thousand. Its economy is based on bubalinoculture, açaí, wood, fishing, and ecotourism (1). Buffaloes are well adapted to hot, humid climates and muddy terrain, but show signs of discomfort when exposed to direct sunlight due to their specific structural characteristics such as dark skin, small number of sweat glands/skin area and thick layer of skin epidermis (2).

It is noteworthy that systems products with the highest degree of animal comfort have added economic and ethical values, and serve a specific class of consumer market (3). Thermal stress imposes behavioral, physiological, and metabolic adaptations to reduce tension and increase animal probability survival, as well as reduce ruminant's performance and compromise animal health (4).

Regarding weather and climate conditions, in years considered as El Niño and/or La Niña, there are mechanisms that modulate atmospheric conditions and promote anomalous changes in the most diverse planet regions, including the thermal-hydro Amazon regime, affecting the monthly, quarterly, seasonal, and annual distribution of rainfall regime, and mean and extreme temperature conditions. Through the UKMO-HadCM3 model it was possible to identify that El Niño events in the 21st century will increase by 20% over the last century, and La Niña tend to intensify their frequency by five times the values observed in the twentieth century (5, 6).

In El Niño years the dry season in Amazon is intensified and this phenomenon is associated to negative anomalies of rainfall that promote river flow (7, 8). Thus, in these extreme event's years, weather conditions can intensify animal discomfort, especially when deprived of shady environments, which can be demonstrated by physiological and behavioral changes. Thus, this work aims to evaluate the effect of thermal-water variables in female buffaloes with and without direct sun exposure in intense El Niño year, through behavior responses and infrared thermography to reinforce the environmental comfort indicators, in Marajó Island, Pará, Brazil.

## Materials and Methods

**Figure 1:**
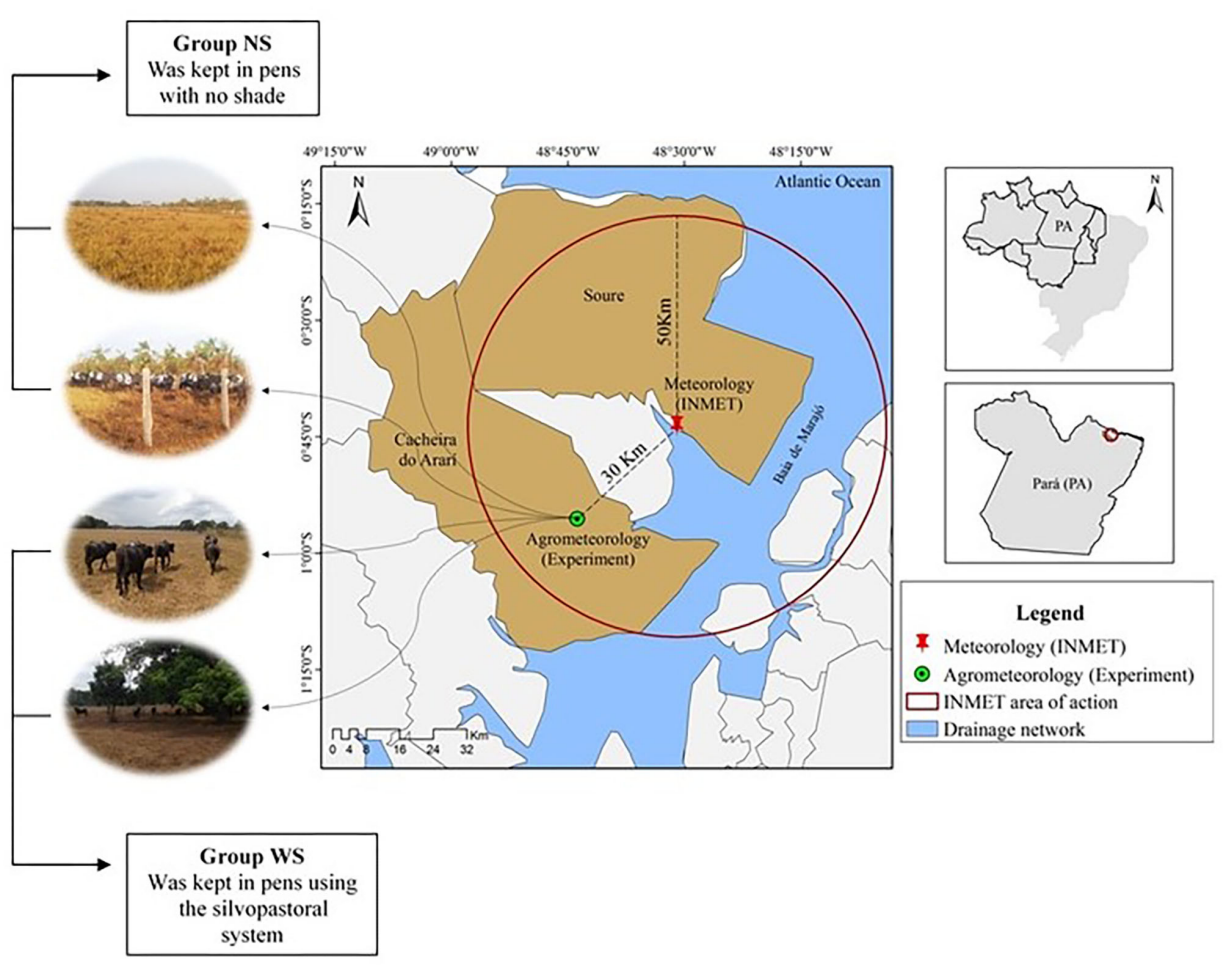
Experimental location area in Cachoeira do Arari municipality, Marajó Island, Pará, Brazil.

During the study, meteorological data were obtained from the meteorological elements of automatic station in Soure, Pará, Brazil (Figure 1). The values are expressed in maximum, mean and minimum air temperature, provided by the National Institute of Meteorology (INMET). Data were also recorded with aid of thermal-hydraulic sensors (HOBO® data logger, model U12−012). They were inserted in agrometeorological mini-shelters and installed in the experimental site, such as air temperature (AT) and relative air humidity (RH). The wind speed (WS) was monitored with aid of a portable digital anemometer, model TAD - 800 (Instrutherm, São Paulo, Brazil).

Twenty Murrah buffaloes, with an average age of 24 months, average weight of 267.92 ± 28 kg, clinically healthy cyclic, non-pregnant and non-lactating females, randomly assigned to two groups (group WS – with shadow and group NS- no shadow). Animal adaptation period to adopted management was 2 days. The WS group (*n* = 10) remained on a 1 ha paddock, in the system with red jamb (*Syzygium malaccense*) and cueira (*Crescentia cujete*) native trees, which allowed about 16% of shade and was measured with tape measure in areas shaded by trees. The NS group (*n* = 10) was kept on paddocks without trees. Both groups were grazing continuously in *Brachiaria humidicola*, with access to drinking water and *ad libitum* mineral salt.

At 6:00 a.m., 10:00 a.m., 2:00 p.m., 6:00 p.m. and 10:00 p.m., for 7 days, animals were taken to the squeeze crush, where they were quickly recorded data from physiological variables that indicate thermal stress: rectal temperature (RT), respiratory rate (RR) and body surface temperature (BST). To obtain the RT, a veterinary clinical thermometer was used, with a maximum scale up to 44°C; it was inserted in the animal's rectum. RR was obtained by inspecting and counting thoracic-abdominal movements for 1 min, with help of a stopwatch. The BST was obtained with aid of an infrared thermometer (Model TD - 965 - Instrutemp, São Paulo, Brazil), activated at a distance of one meter from the measurement points on the animal: forehead, left side of the chest and left flank, using the average of the values.

After 7 days of physiological data collection, buffaloes were changed systems to eliminate animal individual effect. Then, the group that was in the WS system was transported to the NS system and vice versa. After that, animals remained in physiological adaptation again for 2 days in the new system, and then, physiological variables were collected again, for another 7 days.

With physiological variable's data, it was possible to calculate Benezra's Thermal Comfort Index to determine animal's comfort level, using the formula: BTCI = RT/38.33 + RR/23, where: RT is the rectal temperature (°C), and RR is the respiratory rate. Values close to two are considered to be of greater animal comfort, meaning that animals would be presenting ideal RT and RR (9).

With these climatic variable's data, Temperature and Humidity Index (THI) was calculated, proposed by Thom (1959), THI = (0.8 × Average + (Average RH (%)/100) × (Average – 14.4) + 46.4), where T = temperature °C and RH = relative humidity. THI values up to 70 indicate a non-stressful environment, between 71 and 78 critical, between 79 and 83 dangerous, and above 83, an emergency condition.

To more accurately assess the animal's thermal comfort situation, practical Buffalo Comfort Climatic Condition Index (BCCCI) and the practical Buffalo Environmental Comfort Index (BECI) were also calculated using the formulas: BCCCI = 0, 0571 ^*^ RU + 1.0480 ^*^AT (Interpretation: up to 34.65—comfort, from 34.66 to 38.02—danger, from 38.03 to 41.39—thermal stress and above 41.39—emergency) e BECI = 0.8854 ^*^ BST + 0.1695 ^*^ RR (Interpretation: up to 33.55—comfort, from 33.56 to 36.67—danger, from 36.68 to 39.79—thermal stress and above 39, 79—emergency), developed by Silva et al. (10), for buffalo in the Eastern Amazon, Brazil.

**Figure 2:**
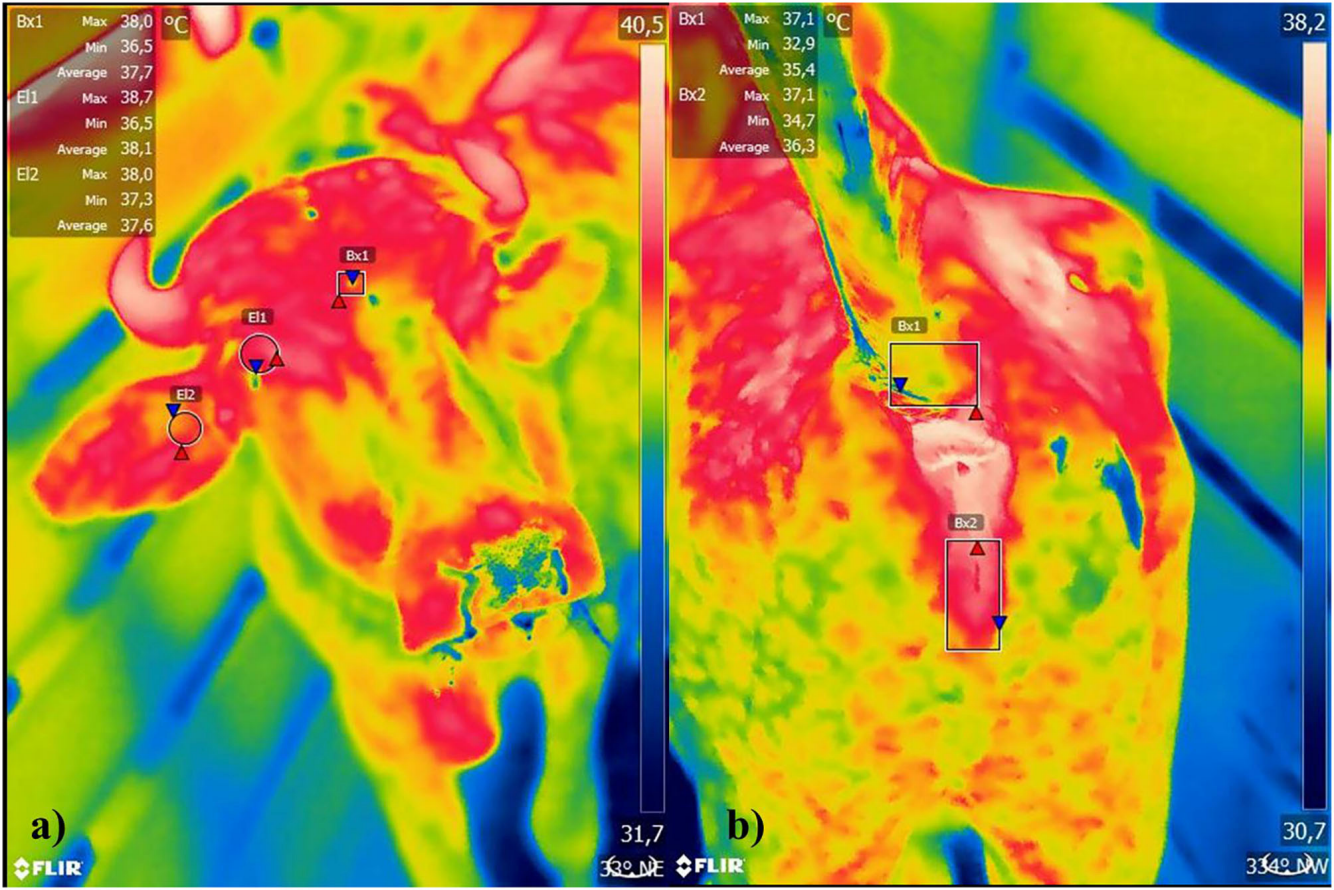
**(a)** Orbital area, ear, **(b)** tail insertion, and vulva of female buffaloes reared in soure, Marajó Island, Pará, Brazil.

Animal's body surface temperatures were also measured using infrared thermography with a FLIR T - series T640bx camera. Animals were evaluated at 10 a.m and 2 p.m., for 1 day, with a total number of 240 images. The camera had autofocus, temperature range −40°C, to 2,000°C, thermal sensitivity <0.03–30°C, spectral scale from 7.5 to 13 μm and optical resolution of 640 × 480 pixels, with emission of 0.98 (11). Each generated thermogram was recorded on a memory card and analyzed by the FLIR Tools software, with thermographic images containing mean, maximum and minimum temperatures, generated from four different anatomical points: orbital area (ORB), ear (EA), tail insertion (TI), and vulva (VUL) (Figure 2), always in that order of collections.

Thermographic image's capture was obtained with animals in crush squeeze, by a single technician, and in the same position, at a distance of one meter. Image's evaluation from ORB and EA areas was limited by a circular trace in the orbital region (12, 13), and in the TI and VUL images, rectangular shape (14).

After physiological variable's collection, animal behavior was evaluated, for 72 consecutive hours, in special files of 60 min each, divided into 12 periods of 5 min, according to the adapted methodology of Lourenço Júnior (15). Females were numbered from 1 to 20, with non-toxic ink, white in color, on the palette and posterior train, to facilitate animal's identification. For evaluation purpose, four categories of activities were considered: grazing (time spent by the animal grazing), rumination), idleness (total time spent by the animal standing or lying) and other activities (walking, defecating, drinking water, urinating and eating salt). The experimental design was completely randomized, considering five shifts of the day: morning (between 6 a.m. and 9:55 a.m.), intermediate (10 a.m. and 1:55 p.m.), afternoon (2 p.m. and 5:55 p.m.), evening (6 p.m. and 9:55 p.m.), late night 1 (10 p.m. and 1:55 a.m.) and late night 2 (2 a.m. and 5:55 a.m.).

The experimental design was completely randomized. For physiological variable's analysis, there was a crossover exchange, in which all animals underwent both treatments, in order to eliminate individual influence, lasting 7 days, and a 2days interval between them, for animal's physiological recovery and then switched treatment, for animal behavior assessments. For thermography, the correlation analysis between the parameters was performed using Pearson's correlation coefficient. The data for physiological (RT, BST and RR), climatic (AT, RU, and WV) and behavioral variables (grazing, ruminating, idleness and other activities) are expressed as means and standard deviations. The statistical treatment was carried out using the SAS v. 9.2 (SAS Institute, Cary, NC, USA, 2010), means being compared using the PROC GLM procedure, with Tukey's HSD test, with 5% probability.

## Results

**Figure 3:**
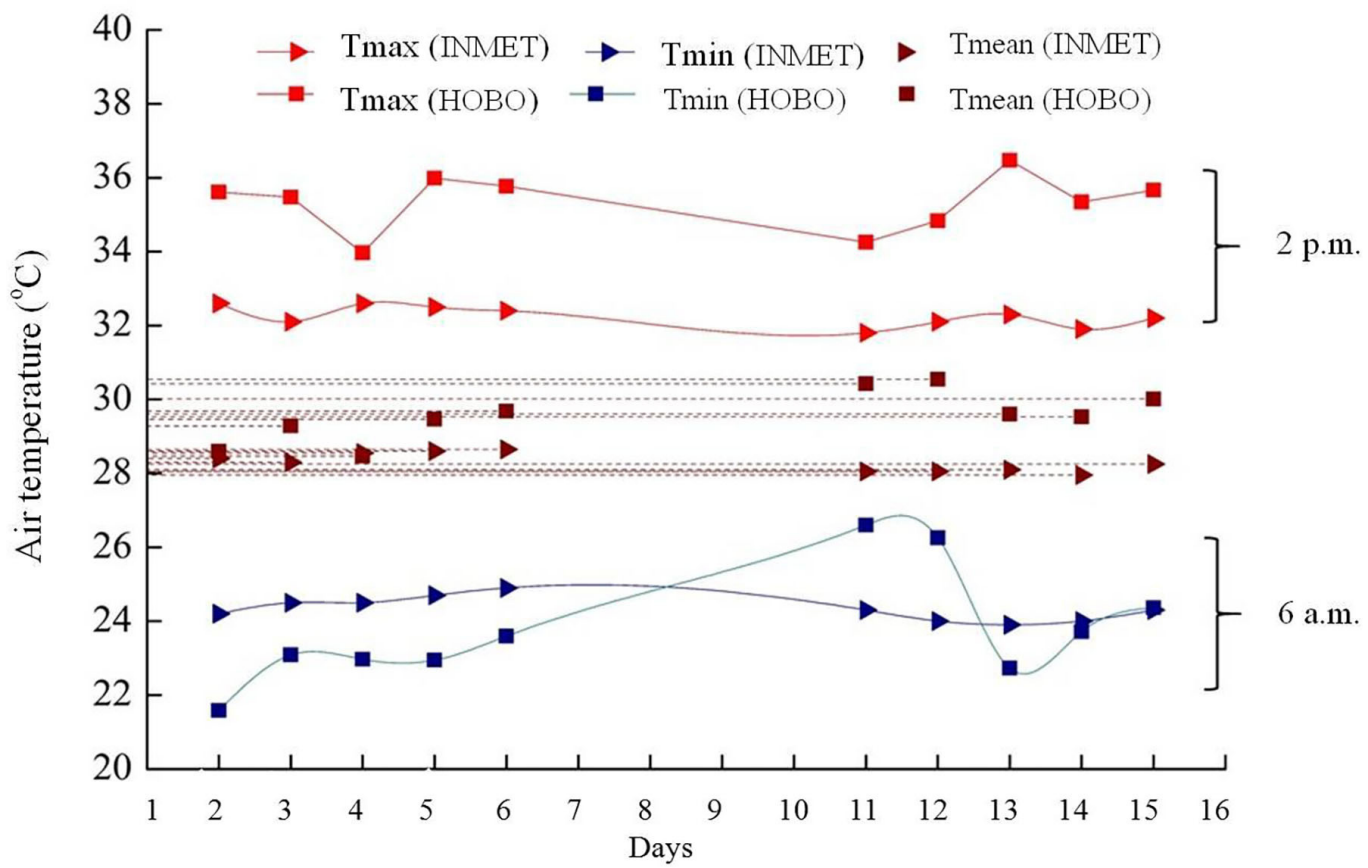
Maximum, mean and minimum air temperature (°C) at 6 a.m. and 2 p.m. of Hobo and weather station located in soure, Marajó Island, Pará, Brazil.

The study area is within the 50 km radius of the INMET weather station (Figure 3). It was observed that the AT in the experimental area had behavior similar (*P* > 0.05) to the automatic surface station, in the months of October and November 2015, at times 6 a.m. and 2 p.m. The Hobo AT at 2 p.m. was higher than that found at the station, probably due to its location near the Atlantic Ocean, wind-receiving area (cyclonal). The mean AT of experimental area over days varied (*P* < 0.05) approximately 2°C in station data. On November 04 and 05, 2015, the late night hours were warmer in the experiment area when compared to the minimum in Soure. These values indicate that during this period there could be greater cloudiness, preventing heat conduction to atmosphere, making the late night warmer in the experimental area.

In the experimental area, the highest mean air temperature was at 2 p.m. in both treatments (34.58 ± 0.90 and 34.96 ± 0.71°C) was observed. There was a significant difference between the groups at 6 a.m., 10 a.m., and 6 p.m., with a variation of approximately 1°C (Table 1).

**Table 1:** Mean values and standard deviation of climate variables at different times in the experimental area, Marajó Island, Pará, Brazil.

**Grupo**	**Air temperature (**^****°****^**C)**				
	**6 a.m**.	**10 a.m**.	**2 p.m**.	**6 p.m**.	**10 p.m**.
NS	24.3 ± 1.87^Eb^	32.6 ± 1.13^Ba^	34.5 ± 0.90^Aa^	29.1 ± 1.13^Cb^	27.1 ± 0.54^Da^
WS	25.1 ± 1.07^Ea^	31.87 ± 1.02^Bb^	34.9 ± 0.71^Aa^	30.1 ± 1.07^Ca^	27.6 ± 0.33^Da^
	**Relative humidity (%)**				
NS	97.1 ± 6.22^Aa^	62.9 ± 4.81^Da^	54.9 ± 3.69^Ea^	74.1 ± 6.0^Ca^	86.4 ± 3.04^Ba^
WS	85.5 ± 3.67^Ab^	61.7 ± 4.40^Ca^	49.7 ± 3.22^Db^	63.6 ± 4.77^Cb^	76.6 ± 1.78^Bb^

*Means within groups with equal capital letters do not differ statistically (P < 0.05). Means at each time, followed by equal lowercase letters, do not differ statistically (P < 0.05). NS, no shadow; WS, with shadow*.

In the experimental area, there was a significant difference between the treatments for RH, at 6 a.m., 2 p.m., 6 p.m. and 10 p.m. (Table 1), except for 10 a.m., where the mean values were higher for the NS group. The highest means in both groups was at 6 a.m. (97.14 ± 6.22 - NS and 85.50 ± 3.67 - WS).

The highest WV means were found at the most critical hours of 10 a.m., 2 p.m. and 6 p.m. (Table 2), according to the Beaufort scale, the winds are moderate breeze at 10 a.m. and 2 p.m., and weak breeze at 6 p.m.

**Table 2:** Wind speed (ms^−1^), mean and standard deviation, at different times, in the experimental area, Marajó Island, Pará, Brazil.

**Time**	**Mean (ms^**−1**^)**	**Beaufort scale**
6 a.m.	0.23 ± 0.71^B^	Calm
10 a.m.	5.61 ± 3.32^A^	Moderate breeze
2 p.m.	5.65 ± 2.90^A^	Moderate breeze
6 p.m.	4.68 ± 2.68^A^	Weak breeze
10 p.m.	1.71 ± 2.16^B^	Light breeze

*Means within the times, with the same capital letter, do not differ statistically (P < 0.05)*.

Regarding physiological variables, the buffaloes showed respiratory behavior changes only at 10 a.m. and 2 p.m. (Table 3), with higher values (*P* < 0.05) of animals from the NS Group (48.07 ± 14.06; 53.49 ± 20.48 vs. 32.76 ± 7.96; 30.30 ± 7.13 mov./min). There was also significant difference for RT (Table 3) between treatments at 10 a.m., 2 p.m., and 6 p.m. (Table 2), whose values were higher (*P* < 0.05) for the animals from NS Group (38.63 ± 0, 50, 39.19 ± 0.59 and 38.65 ± 0.51°C), with the highest mean time at 2 p.m.

**Table 3:** Mean values and standard deviation of physiological variables at different times in the experimental area, Marajó Island, Pará, Brazil.

**Group**	**Respiratory rate (mov./min.)**				
	**6 a.m**.	**10 a.m**.	**2 p.m**.	**6 p.m**.	**10 p.m**.
NS	18.6 ± 3.20^Ca^	48.0 ± 14.06^Ba^	53.4 ± 20.48^Aa^	21.2 ± 4.45^Ca^	18.4 ± 2.76^Ca^
WS	18.5 ± 3.26^Ba^	32.7 ± 7.96^Ab^	30.3 ± 7.13^Ab^	19.8 ± 3.22^Ba^	17.7 ± 3.02^Ba^
	**Rectal temperature (°C)**				
NS	37.1 ± 0.59^Da^	38.6 ± 0.50^Ba^	39.1 ± 0.59^Aa^	38.6 ± 0.51^Ba^	37.9 ± 0.50^Ca^
WS	37.0 ± 0.57^Da^	38.0 ± 0.36^Bb^	38.1 ± 0.45^Bb^	38.3 ± 0.42^Ab^	37.7 ± 0.54^Ca^
	**Body surface temperature (°C)**				
NS	31.4 ± 0.74^Da^	36.1 ± 0.97^Ba^	37.2 ± 1.20^Aa^	32.4 ± 0.74^Ca^	32.3 ± 0.55^Ca^
WS	31.0 ± 0.75^Da^	34.8 ± 0.83^Bb^	35.4 ± 0.93^Ab^	32.1 ± 0.67^Ca^	31.8 ± 0.67^Cb^

*Means within groups with equal capital letters do not differ statistically (P < 0.05). Means at each time, followed by equal lowercase letters, do not differ statistically (P < 0.05). NS, no shadow; WS, with shadow*.

In this study was found that buffaloe's BST in both experimental groups showed significant changes (*P* < 0.05), only at 10 a.m., 2 p.m., and 10 p.m. (Table 3), with values higher than the NS Group (36.17 ± 0 97, 37.20 ± 1.20, 32.32 ± 0.55°C vs. 34.85 ± 0.83 and 35.49 ± 0.93; 31.88 ± 0.67°C), with the largest mean at 2 p.m. and lower at 6 p.m. and 10 p.m. in both treatments.

The environment, in both groups, was critical at 6 a.m., emergency at 2 p.m. and 6 p.m. in danger (Table 4). There was a significant difference between the 10 a.m. treatments, the NS group presented emergency conditions, and WS hazardous environment.

**Table 4:** Mean values and standard deviation of thermal comfort indexes, at different times, in the experimental area, Marajó Island, Pará, Brazil.

	**Temperature and Humidity Index (THI)**				
	**6 a.m**.	**10 a.m**.	**2 p.m**.	**6 p.m**.	**10 p.m**.
NS	75.4 ± 2.81^Ea^	84.0 ± 1.01^Ba^	85.1 ± 1.12^Aa^	80.5 ± 0.99^Ca^	79.0 ± 0.67^Da^
WS	75.7 ± 1.51^Ea^	82.6 ± 0.89^Bb^	84.5 ± 0.89^Aa^	80.5 ± 0.89^Ca^	78.7 ± 0.42^Da^
	**Practical Buffalo Comfort Climatic Conditions Index (BCCCI)**				
NS	31.0 ± 1.71^Ea^	37.8 ± 0.97^Ba^	39.3 ± 0.88^Aa^	34.7 ± 0.89^Ca^	33.3 ± 0.45^Da^
WS	31.2 ± 0.98^Ea^	36.9 ± 0.87^Bb^	39.4 ± 0.69^Aa^	35.2 ± 0.88^Ca^	33.3 ± 0.29^Da^
	**Practical Buffalo Environmental Comfort Index (BECI)**				
NS	30.9 ± 0.84^Da^	40.1 ± 2.77^Ba^	42.0 ± 4.06^Aa^	32.3 ± 1.08^Ca^	31.7 ± 0.70C^Da^
WS	30.6 ± 0.83^Ca^	36.4 ± 1.68^Ab^	36.5 ± 1.67^Ab^	31.8 ± 0.78^Ba^	31.2 ± 0.81^BCa^
	**Benezra's Thermal Comfort Index (BTCI)**				
NS	1.7 ± 0.14^Ca^	3.0 ± 0.61^Ba^	3.3 ± 0.90^Aa^	1.9 ± 0.19^Ca^	1.7 ± 0.12^Ca^
WS	1.7 ± 0.14^Ba^	2.4 ± 0.34^Ab^	2.3 ± 0.31^Ab^	1.8 ± 0.14^Ba^	1.7 ± 0.13^Ba^

*Means within groups with equal capital letters do not differ statistically (P < 0.05). Means at each time, followed by equal lowercase letters, do not differ statistically (P < 0.05). NS, no shadow; WS, with shadow*.

According to BCCCI values, from Silva et al. (10), that was developed for buffaloes raised in the climatic conditions of Eastern Amazon, Brazil; buffaloes raised at Marajó Island also are in thermal stress conditions at 2 p.m., danger at 10 a.m. and 6 p.m. and comfort 6 a.m. and 10 p.m. (Table 4).

BECI was significant (*P* < 0.05) only at 10 a.m. and 2 p.m. (Table 3), with higher values in the NS Group (40.17 ± 2.77; 42.01 ± 4.06 vs. 36.41 ± 1.68; 36.56 ± 1.67), indicating that at 10 a.m. and 2 p.m. the animals in NS group are in emergency conditions and those with access to shade are in danger. In addition, at 6 a.m., 6 p.m. and 10 p.m. the animals in WS group are in comfort.

The BTCI was created to evaluate cattle comfort level and indicates that when values are below 2.0, animals are in greater thermal comfort (9). Buffalo BTCI in both experimental groups indicated significant difference (*P* < 0.05) only at 10 a.m. and 2 p.m. (Table 3); although the values in NS group (3.09 ± 0.61; 3.34 ± 0.90 vs. 2.41 ± 0.34 and 2.31 ± 0.31) were higher, both groups are higher than the considered limit for animal welfare. At other times, and in both groups, the buffaloes remained in a comfort zone. Garcia et al. (16) state that the BTCI has a moderate, positive and significant correlation with the mean air temperature, which justifies the thermal stress at higher temperatures.

Regarding thermographic image's results, Pearson correlation (Table 5) was significant and positive (*P* < 0.01) between RT mean and VUL, TI and ORE mean, maximum and minimum temperatures, and also, with ORB maximum temperature. VUL maximum surface temperature was more correlated (r = 0.84) when compared to eyes, tail insertion and ear.

**Table 5:** Pearson correlation between rectal temperature and surface temperatures measured by infrared thermography in different buffalo anatomic areas at 10 a.m. and 2 p.m., Marajó Island, Pará, Brazil.

	**RT**	**VUL** **mean**	**VUL min**	**VUL** **max**	**TI mean**	**TI** **min**	**TI max**	**EA** **mean**	**EA min**	**EA** **max**	**ORB max**
RT	1.00										
VULmean	0.78^**^	1.00									
VULmin	0.48^*^	0.75^**^	1.00								
VULmax	0.84^**^	0.93^**^	0.57^*^	1.00							
TImean	0.69^**^	0.87^**^	0.63^**^	0.86^**^	1.00						
TImin	0.58^**^	0.75^**^	0.64^**^	0.73^**^	0.86^**^	1.00					
TImax	0.69^**^	0.84^**^	0.62^**^	0.81^**^	0.88^**^	0.81^**^	1.00				
EAmean	0.59^**^	0.61^**^	0.37^*^	0.57^*^	0.44^*^	0.35^*^	0.47^*^	1.00			
EAmin	0.52^*^	0.56^*^	0.32^*^	0.52^*^	0.39^*^	0.32^*^	0.40^*^	0.96^**^	1.00		
EAmax	0.62^**^	0.61^**^	0.37^*^	0.59^**^	0.45^*^	0.37^*^	0.49^*^	0.98^**^	0.92^**^	1.00	
ORBmax	0.65^**^	0.58^**^	0.11^NS^	0.70^**^	0.42^*^	0.33^*^	0.48^*^	0.62^**^	0.58^**^	0.63^**^	1.00

*RT, rectal temperature; VUL mean, vulva mean temperature; VUL min, vulva minimum temperature; VUL max, vulva maximum temperature; TI mean, tail insertion mean temperature; TI min, tail insertion minimum temperature; TI max, tail insertion maximum temperature; EA mean, ear mean temperature; EA min, ear minimum temperature; EA max, ear maximum temperature; and ORB max, orbital area maximum temperature*.

** P < 0001;

* *P < 0.05; NS, not significant*.

**Figure 4:**
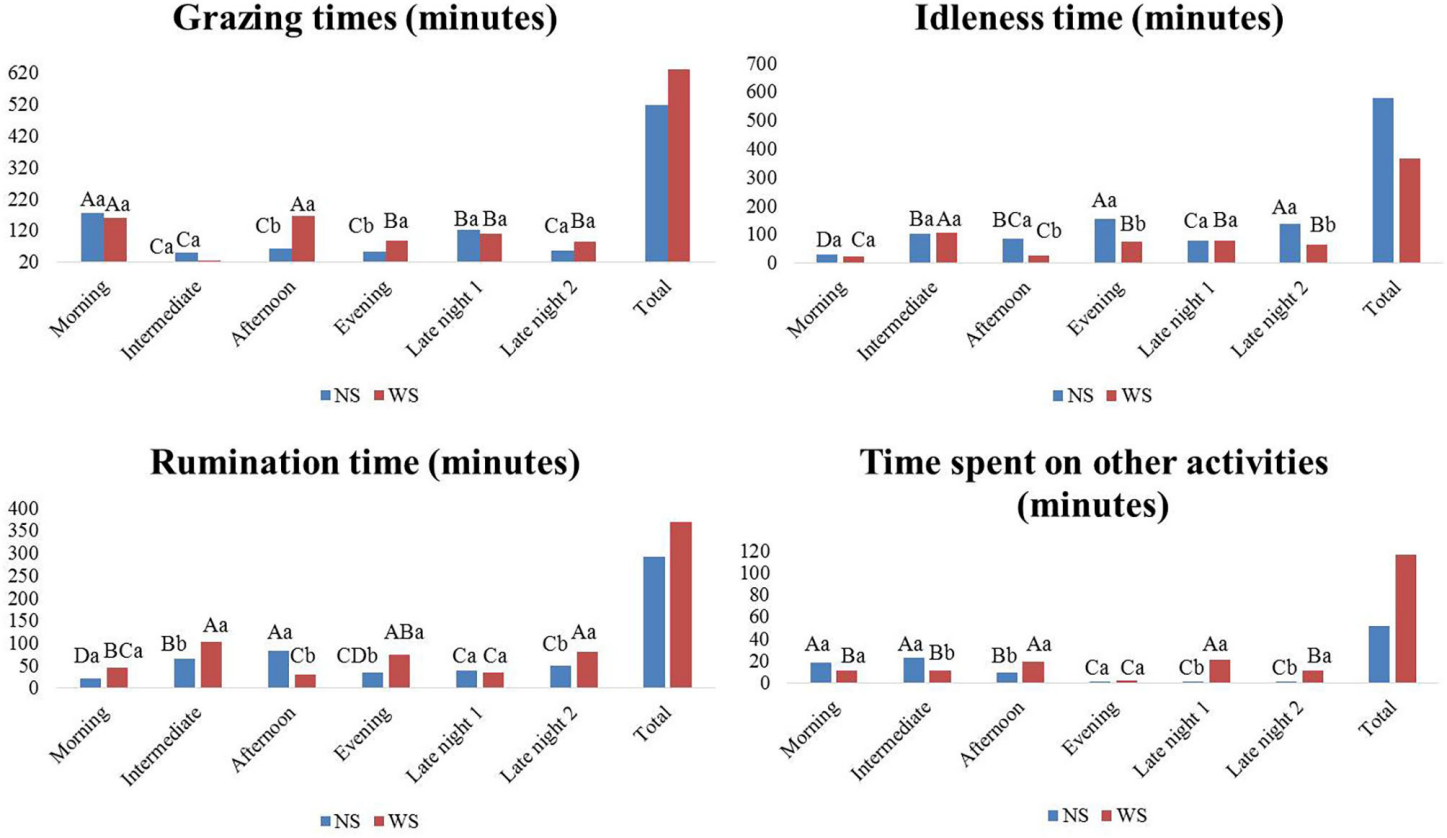
Means of grazing time, rumination, idleness and time spent with other activities, in different shifts, in the experimental area, Marajó Island, Pará, Brazil. Shifts: morning (6:00 a.m.−9:55 a.m.), intermediate (10:00 a.m.−1:55 a.m.), afternoon (2:00 a.m.−5:55 a.m.), evening (6:00 a.m. and 9:55 a.m.), late night 1 (10:00 p.m.−1:55 a.m.) and late night 2 (2:00 a.m.−5:55 a.m.). NS, no shadow; WS, with shadow.

The total time given to grazing was 518.2 min for animals from NS group and 629.5 min for WS group (Figure 4). Group WS intensified grazing in the afternoon and morning, while animals from group NS, the highest average was in the morning.

Rumination was more pronounced in the afternoon shift for NS group, while for shadow group it was the intermediate one, evening and late night 2.

The time spent by animals in idleness was 579.2 min in NS group and 367 min in WS group. Between treatments, there were differences (*P* < 0.05) in the afternoon, evening and late night shifts 2; NS group spent more time in idleness. The longer time is due to the shorter time involved in other activities.

In the morning and intermediate shifts, the animals from NS group obtained the highest means of time spent with other activities, probably intensifying the water intake; since in this shift the environmental situation was severe thermal discomfort. However, during late and early shift 1, WS group stayed longer in other activities.

## Discussion

The present work took place in El Niño year, that was considered the strongest in 65 years, behind only 1982/1983 and 1997/1998 events (17). This phenomenon alters meteorological patterns of certain regions, in weather and climate conditions (18). If the mean rainfall distribution changes to higher or lower values, it may mean that flood or drought thresholds are crossed more often (19).

Differences between the groups at 6 a.m., 10 a.m., and 6 p.m. may impair animal's thermal exchange, especially in the Amazon, which presents air relative humidity. The heat lost to the environment through sensitive heat exchange occurs as a function of temperature differences between body and environment (20).

Air temperature effects are closely linked and dependent on the relative humidity level. This climatic variable is expressed as the relationship between the vapor amounts in the environment that would exist if it were saturated at the same temperature (21).

The relative humidity importance is related to heat loss from evaporation. When the environment is very hot, the low relative humidity favors evaporative mechanisms, which occur faster and cause skin irritation and dehydration. On the other hand, high levels impair heat loss and intensify thermal stress (22). Air temperatures, between 13°C and 18°C; and relative humidity, from 60 to 70%, are suitable for rearing most animals (23). However, these conditions are not observed in the Eastern Amazon, Brazil.

Higher WV means, found at the most critical times of the day, may have contributed to heat loss by evaporation and conduction/convection, as long as air temperature is below the skin temperature. As they grow older, buffaloes suffer a hair natural thinning, avoiding a skin insulating air layer formation, which favors heat dissipation (24).

The animal's ability to withstand heat stress in tropical conditions can be assessed by respiratory rate ranging from 18 to 30 mov./min, rectal temperature from 37.4°C to 37.9°C and body surface temperature, from 25.6 to 35.5 (25).

Changes in respiratory behavior with higher NS group values can be explained due to body overheating, which stimulates the hypothalamus to send neurogenic signals that trigger panting in order to lower the temperature (26). Similar results were observed by Lourenço Júnior et al. (15), with buffalos from Marajó island, in October and November, as the air temperature increased.

Lower RR may be related to the fact that tree shading can be effective in mitigating climate effects in tropical regions. Research in Belém, Pará, indicated that adult buffaloes raised in areas with 18 to 21% shade were protected from direct incidence of solar radiation (16). Thus, RR is a good indicator of thermal load, easy to evaluate in the field (27).

Higher values for NS group animals, with a higher average time at 2 p.m., indicate greater body heat accumulation, higher energy demand for thermolysis and, consequently, less energy available for production (16). Lourenço Júnior et al. (15) observed, in Marajó Island, values that varied from 38.7°C in the less rainy period; while Magalhães et al. (28) also observed the effect of humid tropical climate on buffaloes in the morning and afternoon shifts, 38.05 and 39.26°C, respectively.

Preoptic warming area induces blood vessel's dilatation, which consequently increases BST as blood flows (26). Buffalos still have an anatomical characteristic that differentiates them from other animals, such as thick epidermis and high melanin concentration, making them susceptible to BST elevation when exposed to direct solar radiation in tropics.

THI is considered an indicator to classify environment thermal stress degree (29). When THI exceeds 72, it negatively affects the performance of high-production animals, and can cause death in critically classified dairy cows (30). It also affects somatic cells and total bacterial count in milk (30).

All animals, regardless of rearing system, with or without shade, remained outside their comfort and welfare zone. However, it is worth noting that this index was generated for temperate conditions, making it important to evaluate these data through indices generated for tropics, such as those developed by Silva et al. (10).

Infrared thermography can be defined as a non-invasive technique of thermal mapping a body from infrared radiation normally emitted by the body surface (31). Each animal region emits a different infrared radiation, and it is interpreted as a color, according to the shade scale. Thus, certain areas of the animal's body can be related to body core temperature through the correlation of rectal temperature. In the Pearson correlation, only the maximum surface temperature for the orbital area was used, as it is considered the best representation of rectal temperature for cattle and buffalo (11–13).

Thermography imaging is a tool that is becoming increasingly popular. According to Barros et al. (11), orbital area maximum temperature has the highest correlation with buffalo and cattle rectal temperature, being the most appropriate area to evaluate the animal's thermal conditions, since it suffers interference from the ambient temperature. In addition, Brcko et al. (32) used thermography in several areas of buffalos and verified that eye and cheek could determine thermal stress because these areas are best correlated with the animal's RT. VUL region and tail insertion also appeared to be promising body regions; however, the practical application is questionable because of invasive management for tail occlusion (13).

Accurate monitoring of grazing animal's behavior is essential to ensure health, welfare and sustainable and efficient use of grazing resources. Grazing time is the distribution and magnitude of time devoted to forage seizure; and it involves stages such as, finding and selecting the portion to be ingested, pasture cutting, mouth manipulation and rumination until to be swallowed.

The grazing interval found in this study is within the range found by Fraser and Broom (33), who report that in a period of 24 h, the animal grazes from 4 to 14 h; but there may be variation between beef and dairy animals, their origin (tropical or temperate zone) and rearing system type (free or restricted herd in the field).

Marajó Island livestock is mainly developed in dystrophic soils, resulting in lower quality forage support and productivity. In the less rainy season, the situation worsens, causing significant weight loss in ruminants, which implies longer grazing time as a way to compensate for the poor quality of forage. For Salimos et al. (34), the yield of *Brachiaria humidicola* was always above 1,200 to 1,600 kg DM/ha, regardless of the year period in Marajó Island.

Animals from WS took the opportunity to ruminate the ingested forage in the previous shift, morning, intermediate and late night 1; while group NS reduced metabolic activities, including rumination. Animals prefer to perform rumination activity in cooler periods of the day to compensate for higher internal heat production.

When exposed to high temperatures, animals will no longer eat food and they will be idleness strategically to avoid increased metabolic heat (35).

## Conclusion

- Regardless of whether or not they have access to shade, animals experience thermal discomfort between 10 a.m. and 2 p.m. h;
- Despite environmental heat absorption throughout the day, animals had good physiological recovery, as they presented normal values of physiological variables between 6 and 10 a.m.;
- In the dry period of the year, environmental conditions of Marajó island proved to be a challenge to buffaloes thermoregulation, especially in 2015, an intense year for El Niño, when environmental temperatures were higher than normal;
- Maximum surface temperature of orbital area is the best representation of buffalo rectal temperature;
- Buffaloes kept in a shady tree system graze, ruminate and perform other activities with greater intensity than animals raised in systems without access to shade;
- Planting of trees in pasture areas is indicated to provide shade for the animals, in order to guarantee the well-being, which consequently improves their productive performance.

## Data Availability Statement

All datasets generated for this study are included in the article/supplementary material.

## Ethics Statement

The animal study was reviewed and approved by Ethics Committee from Federal Rural University of the Amazon, protocol number 054/2015 (CEUA) and 23084.013102/2015-01 (UFRA).

## Author Contributions

LA, WJ, and JdS: experiment design. LA, WJ, JdA, RN, and JdS: experiment perform. LA, ABa, JdS, and LM: data curation. ABa and LM: formal analysis. LA, JL, JdA, and ABe: writing-original draft. All authors edited and approved the final manuscript.

## Conflict of Interest

LM was employed by the company Embrapa Eastern Amazon. The remaining authors declare that the research was conducted in the absence of any commercial or financial relationships that could be construed as a potential conflict of interest.
